# Connecting Gerontology and Humanities Through the Legacies of W. Andrew Achenbaum

**DOI:** 10.1093/geroni/igaf122.1258

**Published:** 2025-12-31

**Authors:** Stephen Fogle, James Dowd

## Abstract

Inspired by the legacies of W. Andrew Achenbaum, this symposium features innovative visions for exchange between humanities and gerontological imaginations. With presentations from medical, theological, political, and anthropological disciplines the symposium incorporates contributions that reflect the range of influence that Achenbaum provided during his over four decades of scholarship. Thematically, the symposium moves from reflection to application of Achenbaum’s scholarship. The session begins with a reflective response to Achenbaum’s essay, “Being a Humanistic Patient” to consider what it means to be a Humanistic Clinician. The second paper presents a practical theology of aging called Living Elderhood inspired by Achenbaum’s religion, spirituality, and aging writing. The third paper forwards Achenbaum’s upstanding convictions for democracy through applied theoretical analysis of political narratives and older adults’ political action. The final paper manifests creative potential of incorporating humanities in gerontological research, a cause championed by Achenbaum, through a community engaged research project utilizing Journey Mapping and Graphic Novel-style illustrations of older adults’ health histories. The discussant will provide commentary regarding how the legacies of W. Andrew Achenbaum, as presented by symposium papers, uncover new perspectives, foster collaborations, and address the challenges and opportunities that arise in humanities and gerontological futures. Attendees for this symposium will be able to 1) stimulate provocative conversations about the dynamic ways humanities are manifested in old age 2) garner deeper appreciation of W. Andrew Achenbaum’s legacies, and 3) bring forward new visions of old age to inspire fresh directions of exchange between humanities and gerontological imaginations.

